# Functionalized BODIPYs as Fluorescent Molecular Rotors for Viscosity Detection

**DOI:** 10.3389/fchem.2019.00825

**Published:** 2019-11-26

**Authors:** Wei Miao, Changjiang Yu, Erhong Hao, Lijuan Jiao

**Affiliations:** The Key Laboratory of Functional Molecular Solids, Ministry of Education, Anhui Laboratory of Molecule-Based Materials, School of Chemistry and Materials Science, Anhui Normal University, Wuhu, China

**Keywords:** viscosity, BODIPY, fluorescent molecular rotor, viscosimeter, fluorescent probe, dyes

## Abstract

Abnormal changes of intracellular microviscosity are associated with a series of pathologies and diseases. Therefore, monitoring viscosity at cellular and subcellular levels is important for pathological research. Fluorescent molecular rotors (FMRs) have recently been developed to detect viscosity through a linear correlation between fluorescence intensity or lifetime and viscosity. Recently, 4,4-difluoro-4-bora-3a,4a-diaza-*s*-indacene (boron dipyrrins or BODIPY) derivatives have been widely used to build FMRs for viscosity probes due to their high rotational ability of the rotor and potentially high brightness. In this minireview, functionalized BODIPYs as FMRs for viscosity detection were collected, analyzed and summarized.

## Introduction

**Figure 1:**
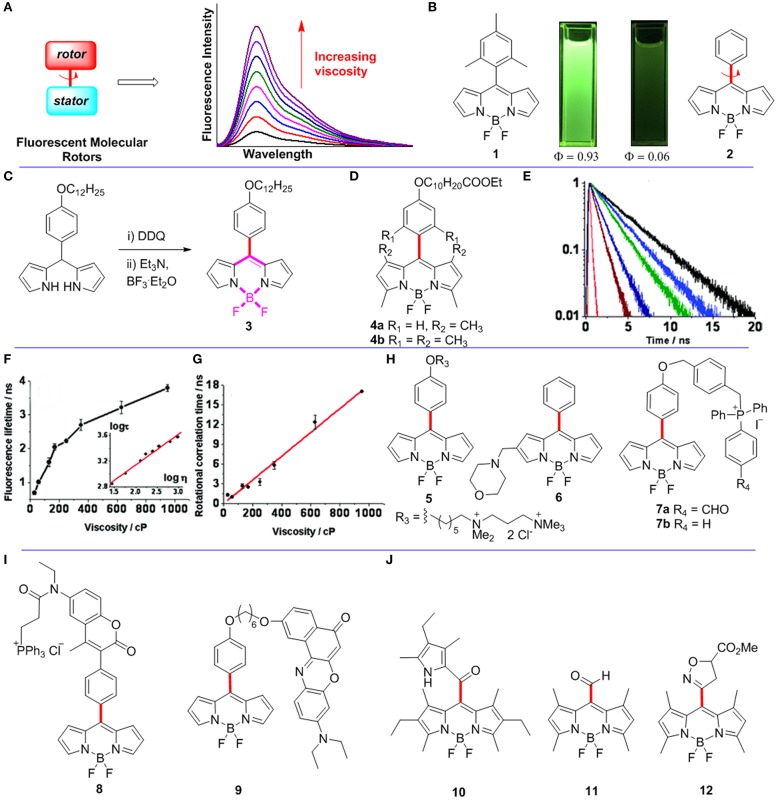
**(A)** The working principle of FMRs. **(B)** Pictures of BODIPYs **1-2** in dichloromethane under 365 nm UV-light irradiation. **(C)** Synthetic route for *meso*-functionalized BODIPY **3**. **(D)** *Meso*-functionalized BODIPYs **4**. **(E–G)** Fluorescence lifetime and rotational correlation time recorded for BODIPY **3** in solvents of various viscosities. Reproduced with permission from Kuimova et al. (2008), Copyright 2008 American Chemical Society. **(H–J)** *Meso*-functionalized BODIPYs **5-12** as FMRs.

The viscosity of cells is an important parameter of the cellular microenvironment, which influences the interaction and transport of biological molecules and signals in living cells (Minton, 2001). Abnormal changes of intracellular microviscosity are associated with a series of pathologies and diseases (Nadiv et al., 1994). Thus, the development of suitable imaging tools to monitor and detect cellular microviscosity is important to study cellular function in both health and disease. Fluorescent molecular rotors (FMRs) are established as tools for monitoring cellular and subcellular viscosity changes because of their high sensitivity, fast-response and non-invasive testing of targets in biological systems. A common feature of FMR is that it consists of two moieties, which are connected by a single bond. One moiety with a large moment of inertia is considered to be fixed, called the stator, and the other moiety with a smaller moment of inertia is called the rotor (Figure 1A). In a low viscosity medium, the rotor rotates freely, and the energy of excitation is dissipated with non-radiative energy. However, in a high viscosity medium, rotation through the C-C bond is constrained, and the excitation energy is released as emission with enhanced fluorescence intensity and lifetime (Uzhinov et al., 2011; Lee et al., 2018). Therefore, the physical mechanism of viscosity dependence of fluorescence quantum yield and lifetime is caused by the steric hindrance of intramolecular rotation. Recently, FMRs have been widely used to measure viscosity of local environment using their changes in fluorescence intensity and lifetime (Kuimova, 2012). From a practical point of view, FMR with high extinction coefficients, long (NIR) excitation wavelength, and potentially high brightness would be desirable (Ning et al., 2017; Hou et al., 2018).

Recently, 4,4-difluoro-4-bora-3a,4a-diaza-*s*-indacenes (BODIPYs) and their derivatives have been paid much attention because of their excellent chemical and physical properties (Loudet and Burgess, 2007; Lu et al., 2014), such as easy functionalization, high molar extinction coefficients, tunable visible to red excitation wavelength, tunable fluorescence quantum yields, as well as excellent photostabilities (Miao et al., 2019; Wang et al., 2019). Not surprisingly, BODIPY derivatives are widely used as imaging probes, fluorescent organic devices, chemical sensors, and as photosensitizers (Cui et al., 2014, 2015; Peterson et al., 2018; Turksoy et al., 2019).

The fluorescence quantum yield of *meso*-(2,4,6-trimethylphenyl)BODIPY **1** is 0.93 in toluene. However, the fluorescence quantum yield of *meso*-phenylBODIPY **2** is only 0.06 in toluene (Figure 1B; Kee et al., 2005). The difference results from free rotations of the *meso*-phenyl group of BODIPY **2** causing energy dissipations as non-radiative. The rotation of its *meso*-phenyl group is constrained by increasing the viscosity of local environment around BODIPY **2**. Therefore, *meso*-phenylBODIPY **2** has the potential to be used as a FMR for viscosity detection. Subsequently, various BODIPY derivatives have indeed been developed as FMRs for viscosity sensors, and the rotor-moiety is mainly attached at the *meso*-position and 2,6-position of the BODIPY scaffold (Dziuba et al., 2016). In this minireview, functionalized BODIPYs as FMRs for viscosity detection are collected, analyzed, and summarized.

### *Meso*-Functionalized BODIPYs as FMRs

In biological systems, changes in viscosity at the cellular level are associated with diseases and pathologies, such as diabetes, infarction, and hypertension. Initially, FMRs were designed to detect cellular viscosity through fluorescence intensity (Haidekker and Theodorakis, 2007). However, measurements based on fluorescence intensity are affected by intracellular uncertainty concentrations. To overcome the problem, Kuimova et al. reported a first *meso*-functionalized BODIPY **3** as a FMR, which detects intracellular viscosity by using fluorescence lifetime instead of fluorescence intensity (Kuimova et al., 2008). As shown in Figure 1C, BODIPY **3** was synthesized through oxidation of the corresponding dipyrromethane with DDQ followed by addition of excess amounts of base and BF_3_.OEt_2_. The authors measured the fluorescence of BODIPY **3** at various viscosities, and indicated that both the fluorescence quantum yield and lifetime (from 0.7 ± 0.05 to 3.8 ± 0.1 ns; Figure 1E) increased dramatically when increasing of the viscosity between 28 and 950 cP. Importantly, the plot of log τ (τ is fluorescence lifetime) vs. log η (η *is* viscosity) was fitted by a straight line (Figure 1F). Control BODIPYs **4a-b** (Figure 1D) were also studied. Since free rotations of the phenyl groups of BODIPYs **4a-b** are restricted, the non-radiative decay process in both dyes is thus prevented. As expected, BODIPYs **4a-b** have fluorescent quantum yields of close to unity in solvents. No apparent viscosity induced fluorescent intensity or lifetime was observed for both **4a** and **4b**. Subsequently, the fluorescence lifetime imaging (FLIM) using **3** was carried out to study intracellular viscosity, and the results indicated that the average viscosity of SK-OV-3 cells was 140 ± 40 cP. Time-resolved fluorescence anisotropy decays with various viscosities were recorded to confirm that the above high viscosity value didn't result from the binding of the rotor to the intracellular targets. The results showed that the rotational correlation time (θ) of BODIPY **3** increased linearly with solvent viscosity (Figure 1G). Finally, the viscosity in SK-OV-3 cells was also measured by using polarization-resolved time correlated single photon counting (TCSPC), and found that the average viscosity of SK-OV-3 cells is 80 cP according to a linear relationship between rotational correlation time θ and viscosity η. The average viscosity of SK-OV-3 cells measured by the two methods is compatible, which shows that the rotor does not combine to intracellular targets. Therefore, the FLIM method using BODIPY based on FMRs is a versatile and also practical method for detecting intracellular viscosity.

Viscosity changes in membranes are associated with various intracellular physiological processes, particularly with various diseases. A few FMRs have successfully detected the viscosity of model lipid bilayers (Hosny et al., 2013; Wu et al., 2013). However, it is difficult to detect viscosity of plasma membranes because of possible effective endocytosis of the probe. For example, BODIPY **3** was only reported to detect viscosity of the lipid membranes of internal cellular organelles. López-Duarte et al. reported a *meso*-functionalized BODIPY **5** to selectively detect viscosity of plasma membranes by adding a double positive charge to the hydrocarbon tail of BODIPY **3** (López-Duarte et al., 2014), which could prevent enucleation and maintain rotor function at the same time (Figure 1H). The fluorescence intensity and lifetime measurements of rotor **5** were studied in solvents of various viscosities. As expected, the gradual increase of the viscosity from 0.6 to 930 cP gave a continuous enhancement of both its fluorescence emission intensity and lifetime. In order to illustrate that this dye is mainly distributed on the cell membrane, the uptake experiments of **5** (8.9 μM) were carried out at 4°C with Mg^2+^ and Ca^2+^ free medium in SK-OV-3 cells. The colocalization results indicated **5** exclusively stained the plasma membranes of SK-OV-3 cells after incubation for more than 30 min. The internal staining of the cells became obvious after incubation time of 55 min. In addition, the FLIM of **5**, measured in SK-OV-3 cells, showed that some staining of internal organelles for an incubation time of 40 min. The lifetimes obtained from this lifetime histogram (40 min) were lower than that obtained after only 10 min incubation. The lifetime histogram for the 40 min image is more adequately fitted with a bimodal Gaussian peak fit, and individual peaks are centered at 1.9 ns (organelles, 200 cP) and 2.2 ns (plasma membrane, 270 cP).

The lysosomal viscosity reflects the microscopic state and function of this organelle. When lysosomal function is impaired, especially through lysosomal storage disease caused by single lysosomal enzymes deficiency, macromolecular substances cannot be decomposed and accumulate in lysozyme. Therefore, it is important to monitor the changes of lysosomal viscosity in real time. In this respect, Wang et al. reported BODIPY **6** with a morpholine moiety at the 2-position of *meso*-phenyl-BODIPY as a FMR to detected viscosity of lysosome by using the FLIM method (Figure 1H; Wang et al., 2013). Morpholine unit was used as an excellent lysosomal localization group according to previous reports (Yu et al., 2012). Indeed, colocalization experiments showed that **6** can selectively strain cell lysosome. At first, they measured the fluorescence intensity of **6** in different pH at a particular viscosity, and revealed that fluorescence intensity of **6** was disturbed by pH changes. In contrast, the lifetimes of **6** at different pH were very similar. Next, they measured fluorescence lifetime of **6** in a series of buffers with different viscosities (from 0.6 to 359.6 cP), and the results showed that its fluorescence lifetimes (log τ) have a strong linear relationship with viscosities (log η). Subsequently, the FLIM of **6** was recorded in MCF-7 cells, and suggested that the average viscosity was ~65 cP in lysosome of MCF-7 cells according to the linear relationship of lifetime against viscosity. Finally, the FLIM of **6** was successfully monitored the dynamic changes of lysosomal viscosity in both dexamethasone and chloroquine stimulated MCF-7 cells.

Mitochondria, as a membrane-bound subcellular organelle, have been found in almost all eukaryotic cells. Mitochondrial viscosity deviations from normal levels will affect the respiratory state of mitochondria, and induce cell dysfunction or even death. Song et al. reported BODIPY **7a** as a novel fixable sensor for detecting mitochondrial viscosity of living cells by the FLIM method (Song et al., 2017). Initially, they synthesized two mitochondrial-localized BODIPYs **7a** and **7b** as showed in Figure 1H. Fluorescence lifetimes (log τ) of both dyes have excellent linear relationships with viscosities (log η) from 0.6 to 360 cP. Moreover, colocalization studies confirmed that both **7a** (Pearson's coefficient 0.92) and **7b** (Pearson's coefficient 0.97) can specially localized in the mitochondria of the SMMC7721 cells. Another set of colocalization imaging experiments of **7a** and **7b** with Mito Tracker Deep Red were recorded under extreme condition (4% formaldehyde solution treatment). Interestingly, **7a** exhibited strong intracellular fluorescence before and after the formaldehyde treatment. In contrast, **7b** only showed strong fluorescence before formaldehyde treatment, however, its fluorescence was remarkably decreased after formaldehyde treatment. These data showed that BODIPY **7a** was immobilized in the mitochondria. Next, the mitochondrial FLIM of BODIPYs **7a-b** suggested that the average viscosity around BODIPYs **7a-b** in the mitochondria of the SMMC7721 cells is 95 and 63 cP, respectively. Similar results were also found in other types of cells, e.g., MCF-7 cells. The authors explained that these different values are due to the different locations of probes: **7a** is mainly immobilized on proteins of the mitochondria, while **7b** is freely distributed in the mitochondria. The larger viscosity value measured by **7a** may be the contribution of the macromolecule of the protein. Finally, the authors further monitored the viscosity changes in abnormal mitochondria (stimulated with rotenone) using BODIPY **7a**. The fluorescence lifetime of BODIPY **7a** was increased from 2.0 to 2.45 ns after stimulated for 8.5 h, and further increased to 2.73 ns after stimulated for 18 h. Thus, **7a**, as a fixable and mitochondria selective FMR, shows a great potential for monitoring mitochondrial viscosity in real time (Zhang et al., 2019).

The second approach for quantitatively determining viscosity through FMRs is based on ratiometric fluorescence measurements. The ratiometric fluorescence probes have a self-calibration effect, which overcomes the uncertainty associated photobleaching, microenvironments, and local probe concentration for conventional fluorescence probes based on intensity changes. Yang et al. reported a fluorescence ratiometry viscosity probe **8** containing a coumarin unit, a BODIPY unit, and a mitochondria selective triphenylphosphonium group (Figure 1I; Yang et al., 2013). The fluorescence changes of **8** in a series of viscosities (from 0.59 to 945.35 cP) revealed that the emission intensities at 427 nm (the emission of the coumarin moiety) and 516 nm (the emission of the BODIPY moiety) both increased with increased viscosity. A strong linear relationship between fluorescence intensity ratio (*I*_516_/*I*_427_) and viscosity (η) was obtained. In addition, fluorescence lifetimes (log τ) also have a good linear relationship with viscosities (log η). According to the above linear relationships, the mitochondrial viscosity in HeLa cells is 62.8 and 67.5 cP, respectively, by using both fluorescence ratiometry and FLIM. Using similar strategy, Yang et al. also reported BODIPY **9 to** detect microviscosity of the endoplasmic reticulum by using both fluorescence ratiometry and the FLIM method (Figure 1I; Yang et al., 2014).

The BODIPY based FMRs described above all contain rotable *meso*-phenyl groups, while BODIPY **4b** (Figure 1) containing substituents on the 1,7-positions of the BODIPY fluorophore is not suitable to be used as FMRs because the *meso*-aromatic group is restricted by the substituents on the 1,7-positions. Thus, it is possible that a less bulky group (than phenyl) on *meso*-position of BODIPY with substituents on the 1,7-positions may allow the rotation in non-viscous media and thus might provide an alternative strategy for designing BODIPY based efficient micro-viscosity probes. Indeed, Yu et al. recently reported a *meso*-2-ketopyrrolyl-derived BODIPY **10** as a new FMR containing substituents on the 1,7-positions (Figure 1J; Yu et al., 2019). The fluorescence lifetime (log τ) of BODIPY **10** has a linear relationship with viscosity (log η). Subsequently, this probe was used to detect viscosity changes during the pathological processes using the FLIM in MCF-7 cells. Moreover, a “distorted-BODIPY”-based viscosity probe **11** with *meso*-CHO group was reported by Zhu et al. (Figure 1J; Zhu et al., 2014). Zatsikha et al. reported a five-membered ring substituted BODIPY **12**, in which the fluorescence intensity (log *I*) has a linear relationship with viscosity (log η) (Figure 1J; Zatsikha et al., 2019).

### 2,6-Functionalized BODIPYs as FMRs

**Figure 2:**
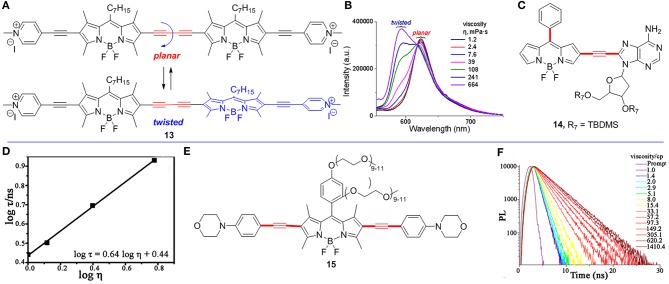
**(A)** Conformational extremes of BODIPY dimer **13**. **(B)** The fluorescence spectra of BODIPY **13** in solvent of various viscosities. **(C)** 2,6-Functionalized BODIPY **14** as a FMR. **(D)** The fluorescence lifetime measurements of BODIPY **14** (3.0 μM) with different viscosities. Reproduced with permission from Li et al. (2016), Copyright 2016 American Chemical Society. **(E)** 2,6-Functionalized BODIPY **15** as a FMR. **(F)** Fluorescence lifetime spectra of BODIPY **15** (0.5 μM at 590 nm) in various viscosities (pH = 1). Reproduced with permission from Li et al. (2018), Copyright 2018 American Chemical Society.

In comparison with the *meso*-functionalized BODIPYs, only a few 2,6-functionalized BODIPYs have been reported to be used as FMRs to detect viscosity. They typically built through the alkyne bridged rotor and BODIPY core. Recently, Zhang et al. reported a ratiometric fluorescence probe **13** with the two BODIPY units linked by butadiyne group (Figure 2A; Zhang et al., 2017). The fluorescence spectra of **13** at different viscosities (from 1.2 to 664 mPa.s) were measured, and its fluorescent emission maximum peaks gradually shifted from 624 to 593 nm with the increase of viscosities (Figure 2B). These two fluorescence emission peaks may be contributed by two extreme conformers of **13** with planar or twisted orientations of the two BODIPY units.

Li et al. reported a RNA-targeted BODIPY **14** as a new FMR to detect intracellular viscosity (Figure 2C; Li et al., 2016). FMR **14** showed two different maximum wavelengths at 496 and 565 nm, respectively. Similar to most fluorescent ratiometry probes, fluorescence measurements of BODIPY **14** showed two different emitted wavelengths (496 and 565 nm), and both fluorescence intensity (log F_565_/F_496_) and lifetime (log τ) have a linear relationship with and viscosity (log η) (Figure 2D). In addition, the colocalization experiments of BODIPY **14** and commercial RNA dye revealed that BODIPY **14** mainly distributed in cytoplasmic RNA (Pearson's correlation 0.96). This result was further supported by imaging of BODIPY **14** in four blood cell types. There was strong fluorescence in reticulocytes (contain RNA), but no fluorescence in red blood cells and other cells (without RNA).

Another FMR **15** (Figure 2E; Li et al., 2018) with two morpholine moieties selectively detected lysosomal viscosity using FLIM. Similar to BODIPY **6**, the fluorescence lifetime (log τ) of BODIPY **15** linearly increased with the increased viscosity (Figure 2F, pH = 1). Moreover, the colocalization experiments indicated that BODIPY **15** mainly distributed in lysosome in Hela cells (Pearson's coefficient 0.95). Next, they monitored the viscosity changes in abnormal mitochondria using BODIPY **15** by treating Hela cells with dexamethasone. Without treating dexamethasone, the histogram of BODIPY **15** indicated the viscosity of the lysosome is 15 cP in Hela cells. However, the viscosity of the lysosome becomes 159 cP after treating with dexamethasone for 1 h according to the linear relationship of lifetime-viscosity.

## Conclusion

In summary, functionalized BODIPYs have recently been developed as novel FMRs for viscosity detection by fluorescence intensity and fluorescence lifetime, in which the rotor-moieties are mainly attached at the *meso*-position and 2/6-positions of the BODIPY scaffold. Those BODIPY based FMRs can be used to detect the subcellular viscosity by introducing a localization group, such as a pyridinium salt, triphenylphosphine salt or morpholine, through fluorescence ratiometry and FLIM methods. By taking advantage of the rapid development of BODIPY synthesis and post-functionalization, we can anticipate that more exciting BODIPY based FMRs decorated with various functional groups with red to near infrared absorption and emission will be developed. BODIPY based FMRs with rotation around other positions (B position, especially) will also be highly anticipated.

## Author Contributions

All authors listed have made a substantial, direct and intellectual contribution to the work, and approved it for publication.

### Conflict of Interest

The authors declare that the research was conducted in the absence of any commercial or financial relationships that could be construed as a potential conflict of interest.
